# Identification of drug-target interaction by a random walk with restart method on an interactome network

**DOI:** 10.1186/s12859-018-2199-x

**Published:** 2018-06-13

**Authors:** Ingoo Lee, Hojung Nam

**Affiliations:** School of Electrical Engineering and Computer Science, Gwangju Institute of Science and Technology (GIST), Buk-gu, Gwangju, 500-712 Republic of Korea

**Keywords:** Drug-target interaction, Machine learning, Protein-protein interaction, Drug-drug interaction

## Abstract

**Background:**

Identification of drug-target interactions acts as a key role in drug discovery. However, identifying drug-target interactions via in-vitro, in-vivo experiments are very laborious, time-consuming. Thus, predicting drug-target interactions by using computational approaches is a good alternative. In recent studies, many feature-based and similarity-based machine learning approaches have shown promising results in drug-target interaction predictions. A previous study showed that accounting connectivity information of drug-drug and protein-protein interactions increase performances of prediction by the concept of ‘guilt-by-association’. However, the approach that only considers directly connected nodes often misses the information that could be derived from distance nodes. Therefore, in this study, we yield global network topology information by using a random walk with restart algorithm and apply the global topology information to the prediction model.

**Results:**

As a result, our prediction model demonstrates increased prediction performance compare to the ‘guilt-by-association’ approach (AUC 0.89 and 0.67 in the training and independent test, respectively). In addition, we show how weighted features by a random walk with restart yields better performances than original features. Also, we confirmed that drugs and proteins that have high-degree of connectivity on the interactome network yield better performance in our model.

**Conclusions:**

The prediction models with weighted features by considering global network topology increased the prediction performances both in the training and testing compared to non-weighted models and previous a ‘guilt-by-association method’. In conclusion, global network topology information on protein-protein interaction and drug-drug interaction effects to the prediction performance of drug-target interactions.

**Electronic supplementary material:**

The online version of this article (10.1186/s12859-018-2199-x) contains supplementary material, which is available to authorized users.

## Background

Drug-target interactions (DTIs) play a key role in drug discovery. Most drugs activate or inhibit the biological functions of a target by binding to the target directly. However, the identification of drug targets by biological and chemical experiments is very laborious and expensive despite the small scale of most experiments [1]. Also, as many drugs are discovered, researchers find that one drug can bind to many targets, and vice versa, which impose systemic approach of DTIs identification [2]. Fortunately, the accumulation of the large-scale of biological and genomic data, such as that in the UniProtKB/Swiss-Prot protein database [3] and the drug data like the DrugBank database [4], allowed researchers to approach DTIs identification via computational and data-driven perspectives.

Therefore, many studies have attempted to predict DTIs by using computational methods to reduce the costs and risks. One remarkable trend is to approach DTIs from a network perspective [5]. Though this strategy has some limitations in that typically only considers the network topology (e.g., interactions or associations of molecules) while utilizing similarities between drugs or proteins as a feature. Yamanashi et al. used bipartite graph models of drug-target pairs in the pharmaceutical space and trained the model with a kernel regression method [6]. With a bipartite graph model integrated on drug and protein space, they reduced heterogeneity between drug and target space. However, because this method undertakes training with a large-scale entire bipartite graph model, high computational power is needed. This computational complexity problem is solved by constructing a bipartite graph per a drug or a target separately, which is called bipartite local model (BLM) [7]. Later, researchers started to utilize more networks to help with predictions. In Chen et al., authors appended protein-protein similarity and drug-drug similarity network onto a bipartite drug-target graph, thus constructing a heterogeneous network. With a random walk with restart on the heterogeneous network, they predicted potential targets and drugs (NRWRH) [8, 9]. However, because this approach constructed the model with only network information, thus incorrect or insufficient information of the network structure could lead inaccurate predictions. Recently, Li et al. used the “guilt-by-association” principle, in which a target protein is likely to interact with a drug if the majority of the protein’s neighbors also do [10]. They constructed a feature-based model and utilized a protein-protein interaction interactome (PPI) network and drug-drug interactions (DDIs) to help prediction by yielding network topology information. With the PPI and DDI, they weighted the features of the drugs and targets using their direct neighbors with respect to edge weights to consider the network topology. This method demonstrated high performance capabilities results from ten cross-validation AUC with a random forest algorithm [11].

However, this approach has a limitation. When integrating the graph topology with features of drug and target, the researchers did not consider the holistic network topology. The “guilt-by-association” principle only took into account the direct neighbors’ information.

**Fig. 1:**
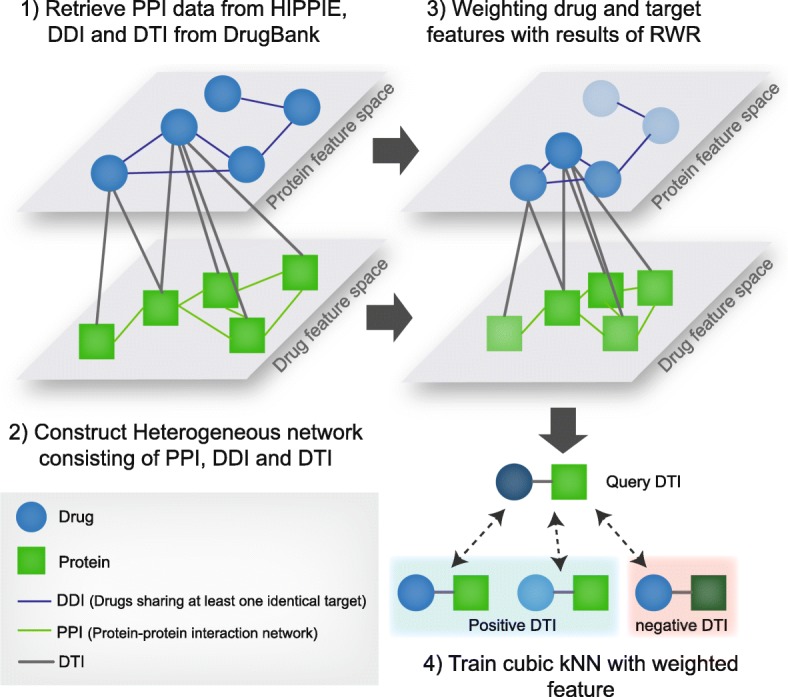
Workflow of the proposed method. 1) We utilized PPI data from the HIPPIE database and DDI and DTI data from the DrugBank database. 2) From both the interactome and DTI data, we constructed a heterogeneous network. 3) We then conducted a random walk with restart algorithm for all nodes (drug and target nodes on each interactome) and weighted features of nodes with the result of the RWR. Weighted features are getting similar on feature spaces for nodes which are closely connected in a network. 4) From the weighted features, we generated positive DTI pair vectors from a bipartite DTI graph and random negative DTI pair vectors. We trained the cubic kNN with the positive and negative DTI pairs

To overcome the limitation, we propose an algorithm capable of considering the global network topology to weight features of drugs and targets by applying the random walk with restart algorithm (RWR) [11]. In this work, we weight the features of drug-target pairs using the random walk with restart algorithm on each interactome network (Fig. 1). First, we utilize PPI information from the HIPPIE database and DTI and DDI information from the DrugBank. Second, we construct three networks, i.e., the PPI network, the DDI network, the DTI network. Third, we transform features of a drug-target pair into a vector format which represents the characteristics of each drug and target. After the transformation process, we run the random walk with restart (RWR) algorithm for all nodes in each PPI network and DDI network separately. With the affinity scores of all nodes for drug and target nodes from the results of the RWR algorithm, we reweight the drug-target feature vectors. Finally, we generate negative drug-target pairs randomly and train a cubic kNN model with the weighted features.

## Methods

### Constructing networks from a database

We constructed three subnetworks, the PPI network, DDI network, and DTI network, which have different sources. First, the PPI network stems from the HIPPIE database, an integrated PPI database consisting of interaction confident data [12, 13]. To construct a more confident PPI network, we used more than 800 interactions to determine an interaction confidence score. As a result, 14,086 proteins and 153,749 PPIs were sourced from the HIPPIE. Second, we constructed the DDI network from the DrugBank [4]. We added edges among drugs if they had the same targets. For 3609 drugs, 77,713 DDIs were constructed. Finally, we constructed the DTI network from the DrugBank, which provides previously known targets of drugs [4]. As a result, 8838 drug-target pairs were sourced. Summarized statistics are shown in Table 1. For training, we built 20 sets of negative samples, which consist of randomly generated 8838 DTI pairs that are not in sourced DTI, but each drug and target are contained sourced drugs and targets.

**Table 1:** Statistics of the training set

Type	Size
Proteins	14,086
Protein-Protein interactions	153,749
Drugs	3609
Drug-Drug interactions	77,713
Drug-Target interactions	8838

### Building independent test dataset

To evaluate the performance of our model in a stringent manner, we created an independent test dataset from the PubChem database [14]. We retrieved positive (active) data from PubChem binding assays, especially using a dissociation constant, and retrieved negative (inactive) data from the PubChem assays except for binding assay types. We consider assays with K_*d*_ ≤ 10*μm* as positive as previous researches did [15] and we treat assays as negative if they’re annotated as inactive. Finally, we collected 6533 positive and 6892 negative DTIs with 629 target proteins and 2635 target drugs.

### Transformation of drug-target features into a vector format

To make DTI understandable by computer and machine learning models, we transformed features of a drug-target pair into a vector. For drugs, we used PaDEL-descriptor [16], which consist of a bit vector with a length of 1024 with the bits representing whether a specific sub-molecular structure exists or not. For proteins, we calculated primary structure descriptors consisting of amino acid compositions (20 dimensions [17], dipeptide compositions (400 dimensions) [18], normalized Moreau-Broto auto-correlations (240 dimensions) [19, 20], Moran auto-correlations (240 dimensions) [21], Geary auto-correlations (240 dimensions) [22], compositions (21 dimensions), transitions (21 dimensions) and distribution (105 dimensions) [23, 24], for a total of 1287 dimensions. Methods to generate drug and target feature vector are summarized in the (Additional file 1 method) . Finally, we concatenated drug vector and target vector to describe a drug-target interaction pair. With the representation of DTIs in a vector format, the characteristics of drugs and target proteins of each pair can be trained for the machine learning model.

### Random walk with restart with DDI-network and PPI-network

To make predictions of DTIs from a network perspective, we yielded affinity scores between the seed node and all nodes using the random walk with restart algorithm (RWR). In the RWR algorithm, starting at the seed node, the random walker diffuses its resources by (1) moving to a neighbor node and (2) restarting from the seed node while restarting probability c. Mathematically, the affinity scores of all nodes during each step are represented by the equation below.

$$ \mathrm{r}=\left(1-\mathrm{c}\right)\overset{\sim }{A}r+ cq $$

Here, q is the starting vector whose seed node s is set to 1 while the others are set to 0, and $\overset{\sim }{A}$ is the normalized adjacent matrix. Consequently, by multiplying the adjacent matrix, it diffuses its resources throughout the network. Moreover, by adding the seed node vector q while to restarting probability c, the method prevents the local accumulation of resources in distant subnetworks. Finally, its resource distribution converges with affinity scores to the seed node with a network topology. By stacking the r_i_ values, the result of RWR for seed node i, we can construct W, the affinity score matrix, whose element W_ij_ refers to how closely node j is connected to seed node i. Because we conducted the RWR with PPI and DDI separately, the W^p^, (N_p_ × *N*_*p*_) affinity score matrix f or proteins and the W^d^, (N_d_ × *N*_*d*_) affinity score matrix for drugs are constructed, where N_p_ is the number of proteins and N_d_ is the number of drugs. Specifically, we conducted the RWR using BEARS, a MATLAB function module [25]. 2.4 Weighting drug-target pair feature vectors by the random walk with restart algorithm.

To consider the network topology in greater detail than in the previous method, we utilize the random walk with restart algorithm, which provides affinity scores for a seed node to all nodes in the network topology. At this stage, we can set the weights of all nodes for the seed node using the affinity scores. Because the resource flow diffuses from 1, the sum of the affinity scores is 1, which means that we do not normalize the weights. As a result, we can easily weight the drug and protein features. For the drug features, they were weighted using the equation below.

$$ {\mathrm{D}}_{\mathrm{i}}=\sum \limits_{i=1}^{N_d}{W}_{ij}^d\times {D}_i $$

For the protein features, the following equation was used.

$$ {P}_{\mathrm{i}}=\sum \limits_{i=1}^{N_p}{W}_{ij}^p\times {P}_i $$

**Fig. 2:**
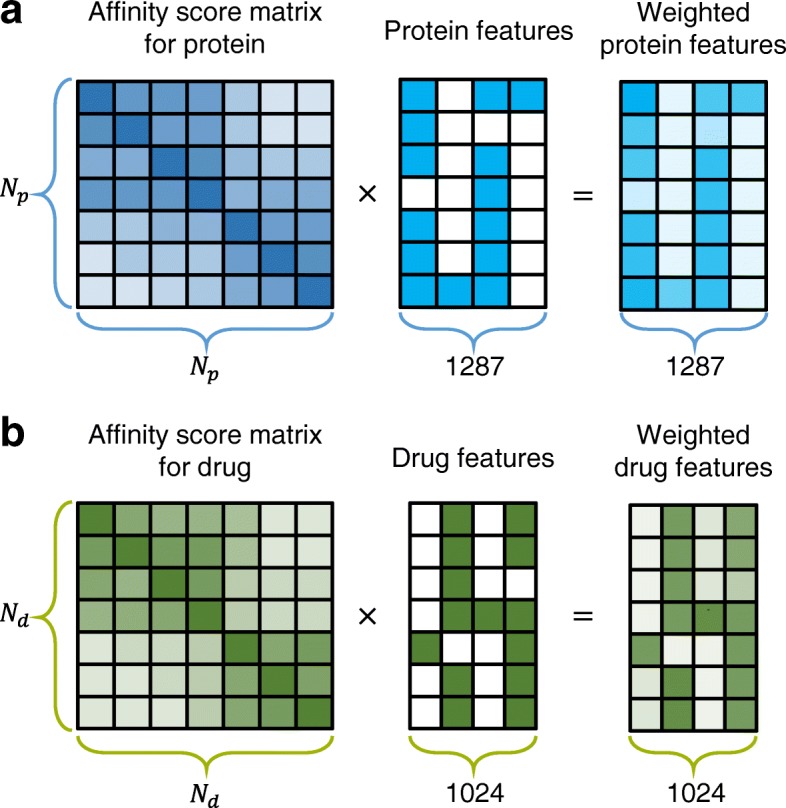
Generation of the weighted features. Weighted features of each drug and target can be constructed by multiplying affinity score matrix from the RWR algorithm with a feature matrix. **a** Protein features. **b** Drug features

Summing up each feature of the nodes to the seed node with the affinity scores from the RWR, we weighted the drug and target feature vector from the network topology. This weighting can be conducted merely by multiplying feature matrix by affinity score matrix as depicted in Fig. 2.

### Weighting features by the RWR algorithm introduce features with the network topology

Weighting with the result of RWR, which introduces the features of other nodes equal to the affinity score, retrieves features with the network topology. Because the RWR algorithm provides affinity scores for all other nodes for a seed node, it will construct a clique from the interactome network, with the node’s value representing the affinity score of the seed node, as shown in Fig. 1 (step 3). By adding the features of the neighbor nodes in the clique to the seed drug and protein features, the proteins and drugs in the subnetwork would show similar features with respect to the network topology. From a biological perspective, we assume that the protein tends to interact with a drug which interacts with the target’s neighbor, and vice versa, in what is termed the “guilt-by-association” principle [10]. We assume that not only the target’s neighbors but also the direct interactors of the neighbors can increase the probability of interaction. In the paper by *Spirin* et al., a gene subnetwork densely connected to others and sparsely connected to others corresponds to (1) complexes and (2) the functional module [26], respectively, where the other members are possible targets. By weighting the features with the RWR algorithm, we can add the information of these members and how close they are to the target protein.

### Training with the weighted features of drugs and proteins

With the weighted feature vectors of the drugs and targets, each known drug-target pair (*d*_*i*_, *p*_*j*_) is represented as a 2311-dimensional vector. To train the machine-learning classification model, we randomly generated drug-target pairs equal to the number of known drug-target pairs, which are assumed not to interact. To standardize our model, we generated 20 negative datasets. We trained the cubic k-nearest-neighbor (kNN) algorithm model with positive data and randomly generated negative data. The kNN algorithm predicts the class of the input vector by selecting the k nearest vectors in the distance kernel. From n vectors, the class with the most frequent representations is predicted as the input vector. For our model, weighting with RWR in each network topology reduces distances on feature space between proteins or drugs which are closely connected in interactome network. Among the many kernels of the kNN algorithm, kNN with the cubic distance metric shows the best performance. In cubic kNN, each element of the distance matrix is defined as

$$ \mathrm{d}\left(\mathrm{x},\mathrm{y}\right)={\left(\sum \limits_{i=1}^n{\left|{x}_i-{y}_i\right|}^3\right)}^{\frac{1}{3}} $$

for different n-dimensional vectors x and y.

The cubic distance maximizes the effect of weighting the features, scaling the difference in every feature.

## Results

### Performance evaluation along restarting probability c and optimizing restarting probability c by the performance of independent dataset

**Fig. 3:**
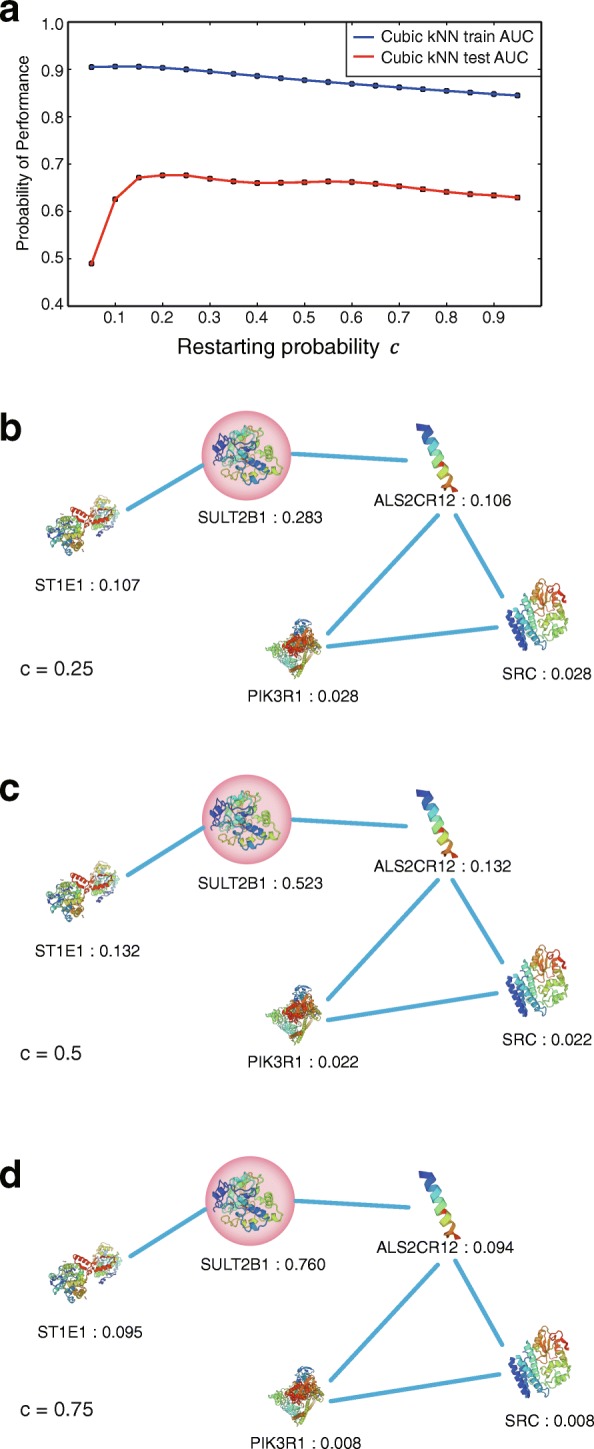
Prediction performances against to the restarting probability c and examples of affinity scores with respect to c. Performances of our model differ from restarting probability c and with different restarting probability c affinity score of neighbor nodes changes. **a** AUC scores from the independent dataset along the restart probability c. Results of the affinity scores of the proteins for the restart probability 0.25, 0.5, and 0.75 in subpanel (**b, c**, and **d**), respectively

The RWR algorithm with seed node i will result in the affinity score row vector r, and the i-th element of r, r_i_, pertains to the restart probability c, as the resource flow will restart with the restarting probability unconditionally. As a result, the weighted features have at least c original features. Therefore, the restarting probability c determines the degree to which the original features of the target proteins or drugs are preserved. Also, it determines how much features of other nodes are retrieved. In Fig. 3a, in the evaluation of the AUC each training and independent dataset, a low c indicates high performance in the training dataset, although a high c (similar to the original features) and a too low c (different from the original features) denote lower performance with the independent dataset. For the training set, a low c makes the features of training data gather on feature space according to the network topology, for low c makes RWR diffuse its resource more. In this study, we found that the optimized restarting probability was 0.25 according to the performance assessment with the independent dataset (Fig. 3a).

### Affinity scores of neighboring nodes differ along restarting probabilities

The random walk with restart (RWR) algorithm gives the affinity scores of all nodes for the seed node, as the resource flow restarts from the seed node, at least as much as the restarting probability c retains resources in the seed node, which prevents local accumulation in distant a subnetwork. As a result, RWR gives high scores for nodes that are closely connected to the seed node and low scores for further nodes regardless of their subnetwork topology. Unlike the ‘guilt-by-association’ principle, it considers not only the target’s neighbors but also further nodes. In Fig. 3b, we queried the target nodes whose affinity scores exceed 0.01, as a result of the RWR with seed node SULT2B1. SULT2B1 has two neighbors, ALS2CR12 and ST1E1, in the network topology. In addition to the neighbors, it shows two additional nodes, SRC and PIK3R1, which are not direct neighbors to the target but which create a clique with ALS2CR12. We examined the affinity scores of these nodes with a various restarting probability c. Figure 3b shows the affinity scores for the queried nodes when c = 0.25. In Fig. 3c, where restarting probability is 0.5, affinity score for seed nodes, increased as 0.5, while affinity scores of other nodes decrease. It means that the relative affinity scores of seed node increase along restarting probability c. In another word, low restarting probability ensures to bring neighbors’ feature more by topology. Similarly, when c = 0.75, weighting considers the seed node’s features rather than the network topology, as we can see in Fig. 3d.

### Weighting features by RWR on network acts as feature extraction

**Fig. 4:**
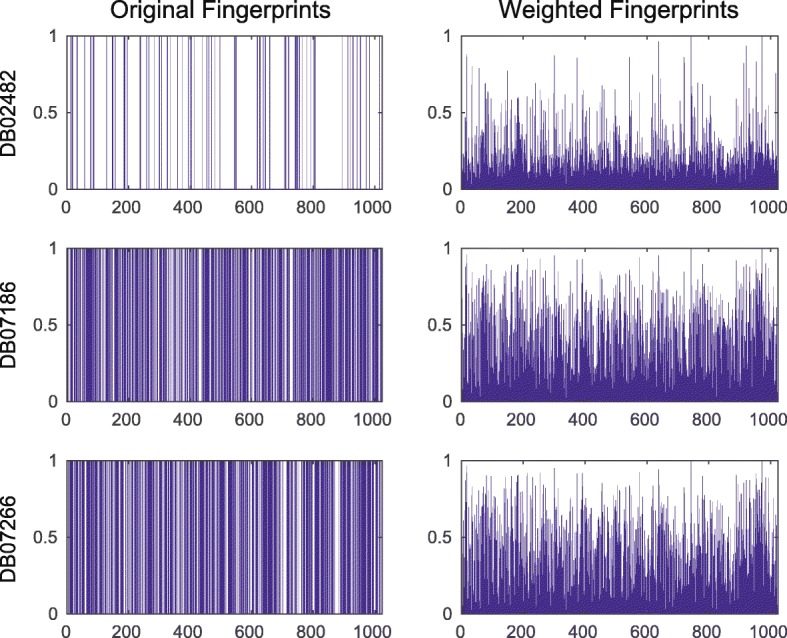
Weighting effects on drug feature by RWR. The left column shows the original fingerprints of drugs, which is a binary vector format. The right column shows fingerprints weighted by affinity scores generated from the RWR with each drug as a seed node

To examine how weighted features by RWR on interactome network gives better performance than other prediction models, we choose three drugs DB02482, DB07186, DB07266, which bind to Aurora kinase A (O14964, AURKA_HUMAN, AUKA). In the DDI network, they are connected to each other because they share the same target AURKA. We first visualized fingerprints of these drugs as shown in the left column of Fig. 4. We can confirm that the original fingerprint features of the three drugs are different (0.4271, the average Euclidean distance for feature). However, after weighting the features by RWR with c = 0.25, they become more similar to each other (Fig. 4, right column, 0.1580, the average Euclidean distance for feature). Because the drugs targeting AURKA are fully interconnected each other, weighting by network topology makes some features as higher values those are commonly shared in the drugs. On the other hand, some features become to have lower values if they are not commonly shared by the drugs. As a result, we can assume that features that show high or low value after weighting would play an important role in the prediction of DTIs with the network perspective. In this perspective, our method bringing features of another node as much as affinity score is elaborated way to yield important feature respect to graph topology than previous ‘guilt-by-association’ method.

### Comparison with the previous methods

**Fig. 5:**
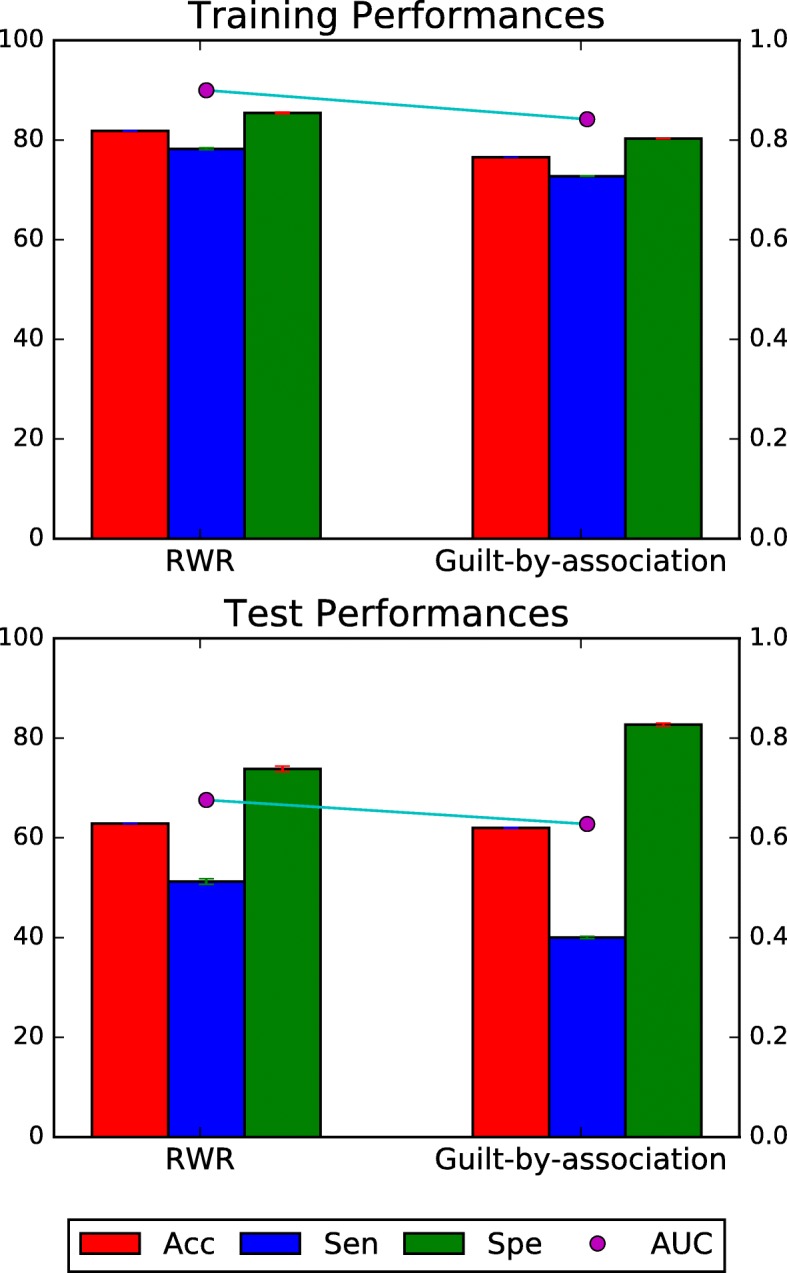
Performance comparison between the previous study and the proposed method. We compared performances of our method with the previous method proposed by *Li etl al*.. Our method shows high performances in the training set overall. In the independent test dataset, our method shows significantly high AUC comparing the previous method

To compare the performance capabilities with those of the previous model, we implement model weighting according to the “guilt-by-association” principle [10]. Overall performance evaluation is shown in Fig. 5. In the training dataset, our method shows higher evaluation performances than “guilt-by-association” method for every evaluation type, which means that our method does better weight with the training dataset. In the independent dataset, our method shows higher AUC 0.675(±0.018) while “guilt-by-association” method shows AUC 0.628(±0.026), which gives statistically significant *p*-value 6.17 × 10^−6^ in paired-sample T-test. On the other hand, we compare our model with another previous method, prioritization by Network-based Random Walk with Restart on Heterogeneous network (NRWRH) to show that not only graph topology information but also protein and drug features can improve the prediction performance. NRWRH can predict DTIs only if drugs and targets are seen in the training set. Therefore, we constructed the independent test dataset using DTIs that are not included in the training set but whose drugs and targets are seen in training. As a result, we yield 661 positive DTIs and 781 negative DTIs with 148 drugs and 171 proteins. As a result, NRWRH methods give AUC 0.6127, while our method gives AUC 0.6025. We plotted the receiver operating characteristic curve in (Additional file 2 Figure S1 ). Although NRWRH gives slightly higher AUC than our method, our method has an advantage, being able to predict interactions of new drugs and targets that have not been used in training.

### Newly predicted interactions

We calculated the average prediction scores of the independent test set from the models trained with 20 training sets. The ten highest DTIs, which were not seen in the training set, are shown in Table 2. Interestingly, many kinase-inhibiting drug-kinase protein pairs are predicted with high scores. For example, pazopanib, a kinase inhibitor, has 169 positive DTI pairs in the independent test dataset; in our model, we correctly predicted 168 DTI pairs with scores over 0.8 while threshold to classify whether positive or negative is 0.62. To examine why pazopanib showed target pairs which were predicted well with high scores, we queried pazopanib and the corresponding positively predicted targets using STITCH [27]. STITCH constructs the PPI and DTI from their database and inputs, as shown in (Additional file 2Figure S2). As a result, we note a densely connected subnetwork consisting of the targets of pazopanib, likely why pazopanib and its targets are predicted well. Because targets of pazopanib are densely connected, their features became similar with graph topology, which gives prediction powers to predict pazopanib as a drug for these targets. Also, we predict several kinase inhibitors-kinase protein pairs for the drugs as tozasertib, axitinib, and dasatinib, all with high scores. Furthermore, we examine DTIs that are exclusively predicted by our method compared to the “guilt-by-association” method. For example, positive JNJ7706621- Q9H4B4(PLK3, Polo like kinase 3) drug-target pairs gives a positive result in our method, but previous “guilt-by-association” gives a negative prediction. We examine PPI of Q9H4B4, summarized in (Additional file 3 Table S1). Q9H4B4 has many PPI with high interaction confidence score, which means features of Q9H4B4 are lost while being weighted by averaging nearby features with interaction confident score. However, in our method, features of Q9H4B4 remains as much as restarting probability at least, and remaining resource flows along network bringing features of other nodes. In this perspective, our method is a more elaborated way to weighting features by network topology, being able to control maintenance of original node.

**Table 2:** Ten highest predicted drug-target pairs

Drug	Target (Gene symbol)	Score (Average)	Evidence
Tozasertib	SRPK3	0.959	[28]
desipramine	SLC6A2	0.956	[29]
Axitinib	SRPK3	0.941	[28]
NVP-TAE684	TNK2	0.929	[28]
Tozasertib	MAPK7	0.916	[28, 30]
Topiramate	CA5A	0.915	[31]
Tozasertib	MAP3K12	0.885	[30]
Pazopanib	LIMK2	0.884	[30]
Pazopanib	MYLK2	0.884	[28, 30]
Pazopanib	CDK16	0.884	[28, 30]

## Discussions

There are various methods for selecting an appropriate PPI for establishing a heterogeneous network in a network-based method using PPI data. For example, PPI databases support confidence score because they usually collect PPI data from not only reviewed data but also various sources such as experimental results, computational predictions and literature mining. In this paper, only PPIs with a confidence score of 800 or higher are used, so that future studies can query the PPI network with various PPI selection criteria depending on the reliability. Also, because confidence scores are only used to query PPI network, it could be possible to use confidence score as prior knowledge for yielding affinity in the random walk algorithm, which means probability for a random walker to stop node j starting from i.

Similar to PPI, there are many ways to choose the appropriate DDI to construct heterogeneous network. In this work, we built edges between drugs sharing the same target for DTI prediction. Although there are many DDI databases such as DCDB and DrugBank, it is hard to bring their DDI information for DTI prediction, because their DDIs imply drug combination. Thus, we should remind that selecting appropriate PPI and DDI could be a crucial step for the network-based DTI prediction studies.

## Conclusions

In this work, we gathered PPI, DDI, and DTI data to construct a heterogeneous network. Also, we weighted the features of drugs and targets using the RWR algorithm. Weighted features by the RWR algorithm allowed the utilization of features with respect to a global interacatome network topology, resulting in features getting similar on feature spaces for nodes which are closely connected in a network.

As a result, our model shows increased cross-validation performance compared to the previous model. At the optimized restarting probability, our method shows better performance in AUC with an independent test set with the cubic kNN as compared to the previous method. Finally, we predicted positive DTIs in an independent dataset, with high scores, showing they are closely connected in the interactome network.

## Additional file

Additional file 1: **Supplementary methods.** Describing the generation process of compound descriptors and protein descriptors. (DOCX 22 kb)

Additional file 2: **Figure S1.** Receiver operating characteristic curve of test data with drugs and targets in training data. **Figure S2.** Pazopanib and its targets during a STITCH prediction. (DOCX 7851 kb)

Additional file 3: **Table S1.** Protein-Protein interactions of Q9H4B4 in Uniprot. (DOCX 14 kb)
