# Supplementary material for: Identification of drug-target interaction by a random walk with restart method on an interactome network
Source: BMC Bioinformatics. 2018 Jun 13;19(Suppl 8):208. doi: 10.1186/s12859-018-2199-x (PMC5998759; doi:10.1186/s12859-018-2199-x)
Supplement: Supplementary file 1 — Supplementary methods. Describing the generation process of compound descriptors and protein descriptors. (DOCX 22 kb) [file 12859_2018_2199_MOESM1_ESM.docx]

# Additional file

# Generating compound descriptors

## CDK fingerprint

PaDEL descriptor generate CDK fingerprint by default [1]. Generating CDK fingerprint works by running breadth first search (BFS), starting at each atom in the molecule. As a result, it generates string representation of paths up to length of six atoms. Hash code for generated SMILES like strings are computed, with standard hashing algorithm. With hash code as seed, random number is retrieved by pseudorandom number generator, whose range is $[0, 1023]$. Retrieved random number is used as index in a fingerprint, binary bit vector [2].

# Generating protein descriptors

## Amino acid composition and dipeptide composition

Amino acid composition is fraction of each amino acid in sequence

$$\mathrm{AAC}\left( i \right)=\frac{N_{i}}{N}$$

Where $i$ is amino acid type (A, R, N, …, Y, V) [3]

Dipeptide composition is fraction of dipeptide in sequence

$$\mathrm{DiPC}\left( i,j \right)=\frac{N_{ij}}{N}$$

Where $i,j$ are amino acid type (A, R, N, …, Y, V) and $\mathrm{ij}$ is dipeptide, combination of amino acid [4].

## Autocorrelation descriptors

Autocorrelation descriptors are kind of topological descriptors, which describe molecular connectivity, correlation between two objects respect to their structural and physico-chemical properties.

Autocorrelation properties are normalized as

$$p_{i}^{'}=\frac{p_{i}-\bar{p}}{\sigma}$$

Where $\bar{p}$ is average value of property and $\sigma$ is variance of property.

Autocorrelation basically defined

$$\mathrm{AC}_{l}=\int_{a}^{b} f\left( x \right) \cdot f\left( x+l \right)\cdot dx$$

Moran-broto autocorrelation descriptors, which is also known as autocorrelation of a topological structure (ATS), are defined as

$$\mathrm{AC}_{moran-broto}\left( d \right)=\frac{\sum_{i=1}^{N-d} p_{i}p_{i+d}}{N-d}$$

Where $d=1, 2, \ldots, 30$ is lag that means topological distance [5, 6].

Moran autocorrelation descriptors are written as

$$\mathrm{AC}_{\mathrm{moran}}\left( d \right)=\frac{\frac{1}{N-d}\sum_{i=1}^{N-d} \left( p_{i}-\bar{p} \right)\left( p_{i+d}-\bar{p} \right)}{\frac{1}{N}\sum_{i=1}^{N} \left( p_{i}-\bar{p} \right)^{2}}$$

[7]

Geary autocorrelation descriptors are described as

$$\mathrm{AC}_{\mathrm{geary}}\left( d \right)=\frac{\frac{1}{2\left( N-d \right)}\sum_{i=1}^{N-d} \left( p_{i}-p_{i+d} \right)^{2}}{\frac{1}{N-1}\sum_{i=1}^{N} \left( p_{i}-\bar{p} \right)^{2}}$$

[8]

## Composition, transition, and distribution (CTD) descriptors

Composition, transition, and distribution (CTD) descriptors describe global composition of amino acid property in protein sequence [9, 10]. Composition means fraction of given amino acid type like amino acid composition. Transition describes the frequency with specific property after residue of different property. Description is defined as the chain length within the first, 25%, 50%, 75% and 100% of the amino acids with a specific property are placed.

1. Yap CW: **PaDEL-descriptor: an open source software to calculate molecular descriptors and fingerprints**. *J Comput Chem* 2011, **32**(7):1466-1474.

2. Steinbeck C, Han Y, Kuhn S, Horlacher O, Luttmann E, Willighagen E: **The Chemistry Development Kit (CDK): an open-source Java library for Chemo- and Bioinformatics**. *J Chem Inf Comput Sci* 2003, **43**(2):493-500.

3. Feng ZP, Zhang CT: **Prediction of membrane protein types based on the hydrophobic index of amino acids**. *J Protein Chem* 2000, **19**(4):269-275.

4. Zhang T, Ding Y, Chou KC: **Prediction of protein subcellular location using hydrophobic patterns of amino acid sequence**. *Comput Biol Chem* 2006, **30**(5):367-371.

5. Chou KC, Cai YD: **Prediction of membrane protein types by incorporating amphipathic effects**. *J Chem Inf Model* 2005, **45**(2):407-413.

6. Lin Z, Pan XM: **Accurate prediction of protein secondary structural content**. *J Protein Chem* 2001, **20**(3):217-220.

7. Horne DS: **Prediction of protein helix content from an autocorrelation analysis of sequence hydrophobicities**. *Biopolymers* 1988, **27**(3):451-477.

8. Sokal RR, Thomson BA: **Population structure inferred by local spatial autocorrelation: an example from an Amerindian tribal population**. *Am J Phys Anthropol* 2006, **129**(1):121-131.

9. Dubchak I, Muchnik I, Holbrook SR, Kim SH: **Prediction of protein folding class using global description of amino acid sequence**. *Proc Natl Acad Sci U S A* 1995, **92**(19):8700-8704.

10. Dubchak I, Muchnik I, Mayor C, Dralyuk I, Kim SH: **Recognition of a protein fold in the context of the Structural Classification of Proteins (SCOP) classification**. *Proteins* 1999, **35**(4):401-407.
