# Supplementary material for: Identification of drug-target interaction by a random walk with restart method on an interactome network
Source: BMC Bioinformatics. 2018 Jun 13;19(Suppl 8):208. doi: 10.1186/s12859-018-2199-x (PMC5998759; doi:10.1186/s12859-018-2199-x)
Supplement: Supplementary file 2 — Figure S1. Receiver operating characteristic curve of test data with drugs and targets in training data. Figure S2. Pazopanib and its targets during a STITCH prediction. (DOCX 7851 kb) [file 12859_2018_2199_MOESM2_ESM.docx]

##
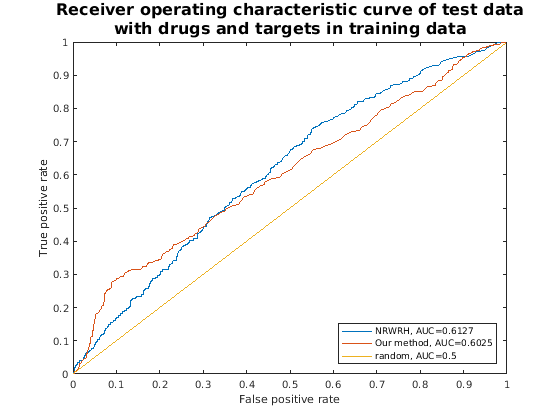
 **Figure S1. Receiver operating characteristic curve of test data with drugs and targets in training data.**

ROC curves for NRWRH method (blue line) and our method (red line) and random(yellow line)

## **Supplementary Figure 2. Pazopanib and its targets during a STITCH prediction**


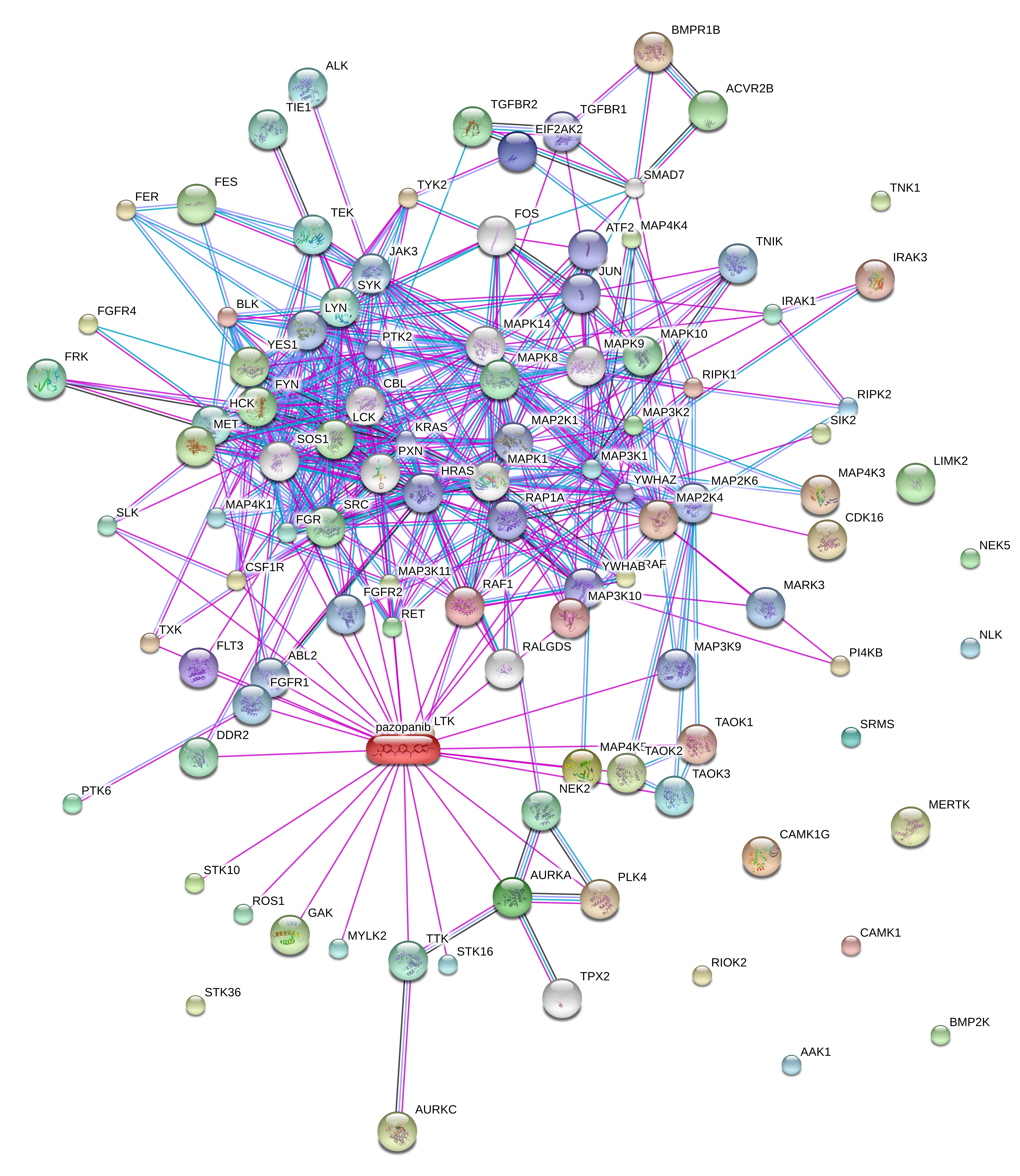


We constructed the PPI and DTI from correctly predicted drug-target interaction. Subnetwork are densely connected, which gives a reason why pazopanib is well predicted.
