# Supplementary material for: Identification of drug-target interaction by a random walk with restart method on an interactome network
Source: BMC Bioinformatics. 2018 Jun 13;19(Suppl 8):208. doi: 10.1186/s12859-018-2199-x (PMC5998759; doi:10.1186/s12859-018-2199-x)
Supplement: Supplementary file 3 — Table S1. Protein-Protein interactions of Q9H4B4 in Uniprot. (DOCX 14 kb) [file 12859_2018_2199_MOESM3_ESM.docx]

## **Table S1. Protein-protein interactions of Q9H4B4 in Uniprot**

| Protein A  (Uniprot ID) | Protein B ID  (Uniprot ID) | Protein A  (Uniprot AC) | Protein B  (Uniprot AC) | Interaction confidence score |
| --- | --- | --- | --- | --- |
| PLK3_HUMAN | SUMO1_HUMAN | Q9H4B4 | P63165 | 0.68 |
| PLK3_HUMAN | PRIO_HUMAN | Q9H4B4 | P04156 | 0.78 |
| PLK3_HUMAN | PTPRC_HUMAN | Q9H4B4 | P08575 | 0.63 |
| PLK3_HUMAN | VRK1_HUMAN | Q9H4B4 | Q99986 | 0.75 |
| PLK3_HUMAN | CENPU_HUMAN | Q9H4B4 | Q71F23 | 0.49 |
| PLK3_HUMAN | SYUA_HUMAN | Q9H4B4 | P37840 | 0.52 |
| PLK3_HUMAN | SYUB_HUMAN | Q9H4B4 | Q16143 | 0.52 |
| PLK3_HUMAN | AURKA_HUMAN | Q9H4B4 | O14965 | 0.56 |
| PLK3_HUMAN | BUB1B_HUMAN | Q9H4B4 | O60566 | 0.68 |
| PLK3_HUMAN | PCH2_HUMAN | Q9H4B4 | Q15645 | 0.63 |
| PLK3_HUMAN | MPIP3_HUMAN | Q9H4B4 | P30307 | 0.9 |
| PLK3_HUMAN | MK01_HUMAN | Q9H4B4 | P28482 | 0.52 |
| PLK3_HUMAN | MP2K1_HUMAN | Q9H4B4 | Q02750 | 0.52 |
| PLK3_HUMAN | CHK2_HUMAN | Q9H4B4 | O96017 | 0.88 |
| PLK3_HUMAN | CIB1_HUMAN | Q9H4B4 | Q99828 | 0.87 |
| PLK3_HUMAN | P53_HUMAN | Q9H4B4 | P04637 | 0.88 |
